# Engineering Properties of PVA Fibre-Reinforced Geopolymer Mortar Containing Waste Oyster Shells

**DOI:** 10.3390/ma15197013

**Published:** 2022-10-10

**Authors:** Ziming Deng, Zhangfeng Yang, Jin Bian, Xinxiang Pan, Guanglin Wu, Fei Guo, Ruizhi Fu, Hongjin Yan, Zijun Deng, Siqi Chen

**Affiliations:** 1 College of Ocean Engineering and Energy, Guangdong Ocean University, Zhanjiang 524088, China; 2 Department of Mechanical, Aerospace and Civil Engineering, School of Engineering, The University of Manchester, Manchester M13 9PL, UK; 3 Beijing Municipal Construction Co., Ltd., Beijing 100045, China; 4 School of Management, Guangzhou College of Technology and Business, Foshan 528135, China

**Keywords:** geopolymer mortar, waste oyster shells, PVA fibre, engineering properties

## Abstract

Recycling crushed waste oyster shells (WOS) as a fine aggregate is an attractive method of disposal. However, its use in geopolymer mortar has not been reported. The influence of PVA fibres on the engineering properties of the new geopolymer mortar is still unclear. To bridge the gap, this study investigated the influence of various PVA fibre contents (0–1.05 vol%) on the flowability, compressive, flexural strengths, drying shrinkage, sorptivity, chloride resistance, porosity, fibre dispersion, embodied CO_2_ emissions (ECO_2_e), and embodied energy (EE) of the geopolymer mortar. The results indicated that the inclusion of 0.15–1.05 vol% of PVA fibres improved the flexural strength by 10.10–42.31% and reduced the drying shrinkage by 13.37–65.79%. The flowability and compressive strength decreased by 10.78–34.28% and 7.50–27.65%, respectively, but they were sufficient for construction. The sorptivity increased by 1.45–15.16%, and the chloride resistance decreased by 15.09–56.35%, but the geopolymer mortar was still classified as low chloride penetrability. In summary, the optimal content of PVA fibres is 0.45 vol%, and the geopolymer mortar has good engineering properties and eco-efficiency. The cost analysis and high-temperature resistance of the geopolymer mortar are neglected in this study, which should be evaluated in future work.

## 1. Introduction

Oysters are a common aquatic product. In 2010, China’s oyster shell production reached 5 million tons [1]. The global quantity of waste oyster shells (WOS) is about 200,000 million tons per year [2]. However, a tiny fraction of the enormous WOS is recycled (e.g., fertilisers and handicrafts), and most of the waste is buried in landfills or discarded in fields, which causes odours and sewage, contaminates soils, and breeds mosquitoes and flies [2,3]. It is necessary to recycle WOS in a sustainable way.

Recycling industrial and natural wastes to produce sustainable construction materials has attracted a lot of attention for a decade [4,5]. For instance, palm kernel shells [6], palm oil fuel ash [7], recycled concrete [8], rubber tires [9], and recycled asphalt [10] are used as alternatives for aggregates. Crushed WOS also have a lot of potential as an aggregate in cement mortar and concrete. [1,3,11,12]. WOS aggregates have the following benefits of sustainability relative to river sand. First, the excessive mining of river sand causes the instability of the riverbed and increases drought risk [13]. The partial replacement of river sand with WOS aggregates reduces the consumption of river sand and mitigates the risk. Second, the use of WOS aggregates reduces the energy consumption and CO_2_ emissions caused by the mining of river sand. Third, recycling WOS as a fine aggregate prevents odours, sewage, and soil contamination caused by its discarding.

On the other hand, wheat straw ash [14], oil shale ash [15], silica fume [16], and kaolin deposits [17] are used to partially replace ordinary Portland cement (OPC). However, the production of OPC causes high CO_2_ emissions and energy consumption, which is caused by the de-carbonation reaction of limestone at 1400–1500 °C [18]. Geopolymer is a sustainable and feasible alternative for OPC. Industrial wastes, granulated blast-furnace slag (GBFS) and fly ash (FA) are often used to produce geopolymer via the alkali-activated reaction (AAR). Geopolymer has been proved to perform better than OPC in terms of mechanical properties [19,20] and durability [21,22].

Geopolymer mortar is known to have higher drying shrinkage than cement mortar. Brittleness is another disadvantage of geopolymer mortar. Since PVA fibers are significantly stable in alkaline environments and have a higher elastic modulus than other fibers [23], previous studies have proposed the inclusion of polyvinyl alcohol (PVA) fibres to minimise these two drawbacks. For instance, Xu et al. [24] stated that the inclusion of 0.5–2.0 vol% of PVA fibres significantly improved the toughness of geopolymer composites, but the optimal content of PVA fibres was not revealed. Zhang et al. [25] stated that 0.8 and 1.2 vol% of PVA fibres exhibited the highest compressive and flexural strengths of geopolymer mortar containing 100% river sand, respectively. However, the influences of these two contents on chloride resistance, sorptivity, porosity, and fibre dispersion were not investigated. In similar studies [26,27,28], the optimal content of PVA fibres was also neglected in terms of these properties.

In addition, Xu et al. [29] stated that the optimal content (0.4 vol%) of PVA fibres significantly controlled the drying and autogenous shrinkage of geopolymer mortar containing 100% river sand. However, the influence of this content on other durability was not reported. Wang et al. [30] and Al-mashhadani et al. [31] also stated that the inclusion of 0.4, 0.8, 1.0, 1.2, 1.5, and 2.0 vol% of PVA fibres decreased the drying shrinkage of geopolymer mortar containing 100% river sand. However, chloride resistance, sorptivity, porosity, and fibre dispersion were not investigated in these studies.

Previous studies have focused on the mechanical properties and drying shrinkage of the conventional PVA fibre-reinforced geopolymer mortar containing 100% river sand. The use of WOS to replace river sand has also been reported in cement mortar. However, recycling WOS aggregates in PVA fibre-reinforced geopolymer mortar has not yet been revealed. Due to the lack of experimental evidence, the influence of PVA fibres on the mechanical properties and drying shrinkage of the new geopolymer mortar is still unclear. Moreover, the chloride resistance, sorptivity, eco-efficiency, porosity, and fibre dispersion of the new geopolymer mortar are also unknown.

To resolve these uncertainties, this study investigated the influence of different PVA fibre contents (0, 0.15, 0.30, 0.45, 0.60, 0.75, 0.90, and 1.05 vol%) on the flowability, compressive, flexural strengths, drying shrinkage, sorptivity, chloride resistance, porosity, fibre dispersion, embodied CO_2_ emissions (ECO_2_e), and embodied energy (EE) of the geopolymer mortar containing WOS aggregates. This study first time reports the engineering properties and eco-efficiency of the new geopolymer mortar. It promotes the recycling of WOS and results in sustainable construction materials.

## 2. Materials and Methods

### 2.1. Materials

As precursors (aluminosilicate materials), commercial GBFS and FA (Class F) were employed. They were sourced from Huiding Environmental Technology Co., Ltd., China. Table 1 shows their chemical compositions. The basicity coefficient of GBFS was 0.96, and its hydration modulus was 0.90. In addition, Table 2 lists the mechanical and physical characteristics of PVA fibres. The PVA fibres were sourced from Tianyi Engineered Fibres Co., Ltd., Jintan City, China.

**Figure 1:**
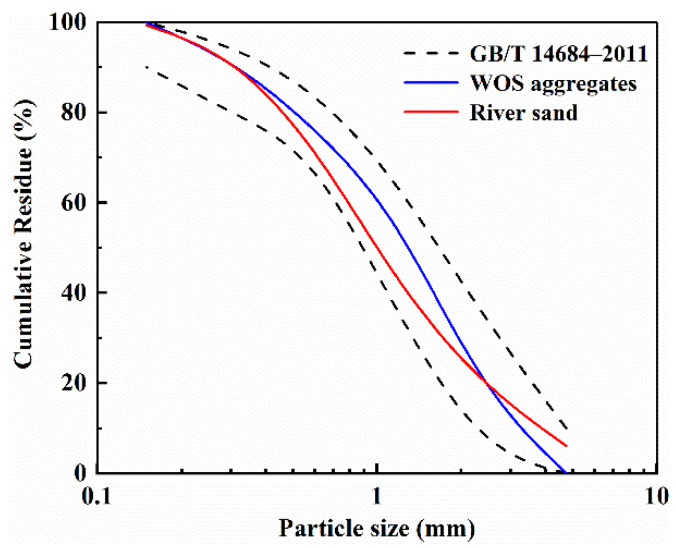
Gradation curves of WOS aggregates and river sand.

As fine aggregates, crushed WOS and river sand were used in the geopolymer mortar. The WOS was sourced from a local landfill. Discarded oyster shells were crushed to produce WOS aggregates, and undesirable wastes and organics were removed. The gradation curves of river sand and WOS aggregates are shown in Figure 1. According to GB/T 14684-2011 [32], they met the gradation requirements for construction sand.

Alkaline activators were the mixed NaOH and Na_2_O·2.25SiO_2_ solutions. They were sourced from Huiding Environmental Technology Co., Ltd., China. The NaOH solution was fabricated using NaOH pellets with a class of analytical reagents, and its concentration was 8 mol/L. The commercial sodium silicate solution had 56.26% of H_2_O, 29.99% of SiO_2_, and 13.75% of Na_2_O.

### 2.2. Mixing, Casting, and Curing

Table 3 shows the mix proportions, and they were based on the references [33,34]. The PVA fibre content was 0, 0.15, 0.30, 0.45, 0.60, 0.75, 0.90, and 1.05 vol%. The ratio of NaOH to Na_2_O·2.25SiO_2_ was 0.5, and the two solutions were mixed and cooled. The ratio of activators to precursors was 0.6, and that of fine aggregates to precursors was 1.6. The replacement ratio of river sand with WOS aggregates was 20%, which was recommended by Liao et al. [1] and Liu et al. [3].

The prepared GBFS, FA, and fine aggregates were pre-mixed for 3 min, and the activator solution was then added. The PVA fibres were dispersed into the mixture, which was followed by another 4 min of mixing. Surface-oiled moulds were filled with the resulting fresh mixture and vibrated for compaction. The specimens were cured until test ages at 20 ± 1 °C and 60 ± 5% relative humidity.

### 2.3. Test Methods

#### 2.3.1. Flowability

The flowability of the mixture was investigated using the flow table test based on GB/T 2419-2005 [35]. The mixture was manually compacted based on the procedures in the code. Immediately after the flow table jumped and dropped 25 times in 25 ± 1 s, the flow mould was removed via vertically lifting. The average of the two perpendicular diameters of the spread (collapsed) mixture was determined as the flowability.

#### 2.3.2. Strength

**Figure 2:**
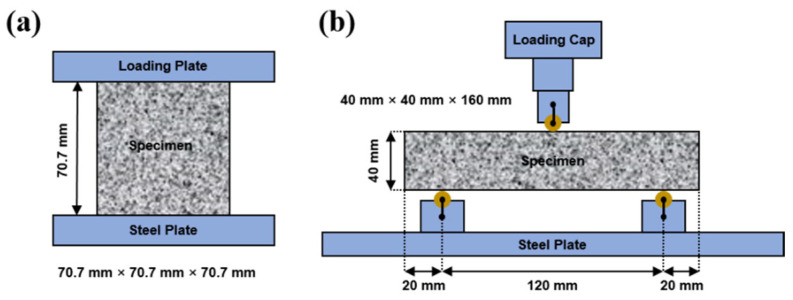
Schematics of the tests for compressive (**a**) and flexural (**b**) strengths.

The tests for compressive and flexural strengths were conducted based on JGJ/T70-2009 [36] and JTG E30-2005 [37], respectively, using a loading rate of 1.2 kN/s and 50 N/s, respectively. Schematics of the tests are shown in Figure 2. The specimens used for the compressive and flexural strength tests had dimensions of 70.7 mm × 70.7 mm × 70.7 mm and 40 mm × 40 mm × 160 mm, respectively. The specimens were aged for 1, 7, 14, and 28 d before testing. For each mix proportion, triplicate specimens were used in these tests.

#### 2.3.3. Drying Shrinkage

Drying shrinkage was determined according to JGJ/T 70-2009 [36]. The dimension of specimens for this test was 40 mm × 40 mm × 160 mm, and the number of specimens for each mix proportion was three. Their initial lengths and length changes were recorded using a length comparator. The test periods were 1, 3, 5, 7, 10, 14, 21, and 28 d. The drying shrinkage was calculated using the following equation:

(1) $$\varepsilon_{t}=\frac{L^{0}-L^{t}}{L-L^{d}}$$

where ε*_t_* is the values of drying shrinkage at 1, 3, 5, 7, 10, 14, 21, and 28 d (%); *L*_0_ is the initial length of each specimen immediately after de-moulding (mm); *L_t_* is the measured length of each specimen at 1, 3, 5, 7, 10, 14, 21, and 28 d (mm); *L* is the nominal length (160 mm); *L_d_* is the summation of the inserted depths of two copper probes (mm).

#### 2.3.4. Sorptivity

**Figure 3:**
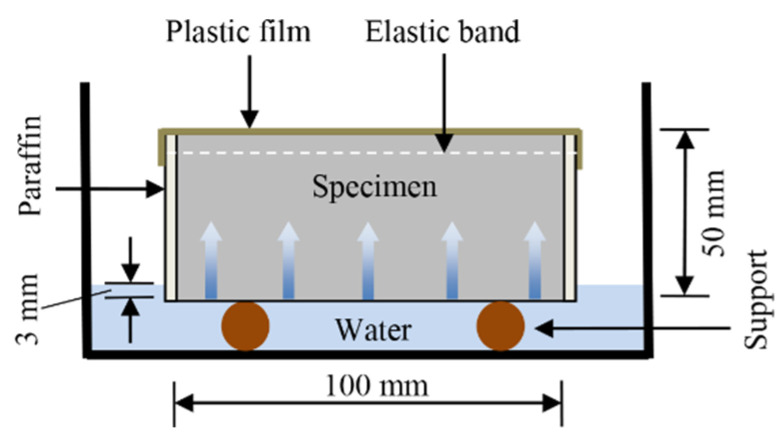
Schematic of the sorptivity test.

To investigate sorptivity according to ASTM C1585-20 [38], disc-shaped specimens of 100 mm in diameter and 50 mm in height were employed. Figure 3 shows the schematic of the test. The specimens were dried at 50 ± 1 °C until they reached a constant mass. The specimens’ curved surfaces were then sealed with paraffin, and a plastic film was applied to their top surfaces. Immediately after exposure to water, the cumulative masses of the treated specimens were recorded at 60 s, 5 min, 10 min, 20 min, 30 min, 1 h, 2 h, 3 h, 4 h, 5 h, and 6 h. The test ages of sorptivity were 7 and 28 d. For each mix proportion, triplicate specimens were used in the test.

#### 2.3.5. Rapid Chloride Penetration (RCP)

**Figure 4:**
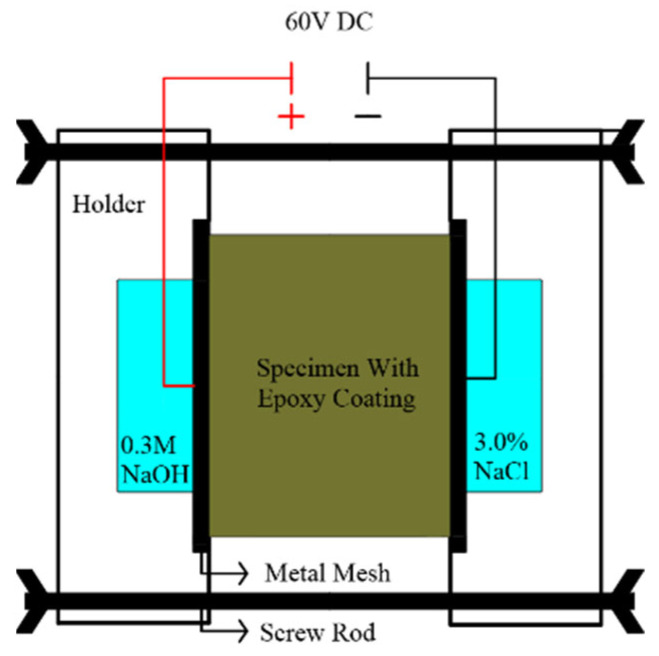
Schematic of the RCP test.

To investigate RCP based on GB/T 50082-2009 [39], disc-shaped specimens of 100 mm in diameter and 50 mm in height were employed. RCP’s test ages were 7 and 28 days. Figure 4 shows the schematic of the RCP test. A vacuum water retention of the specimens was conducted, and the curved surfaces of specimens were sealed by epoxy. A DC voltage of 60 V was used, and the anode and cathode cells were filled with 0.3 mol/L NaOH and 3.0 wt% NaCl solutions, respectively. The charges passed through the specimens were recorded for 6 h at an interval of 30 min. The total charges passed were calculated and used to evaluate chloride resistance. Three triplicate specimens were used for each mix proportion in the test.

#### 2.3.6. Porosity

To evaluate the porosity of samples, mercury intrusion porosimetry (MIP) was performed The samples were cut from the mortar specimens using a diamond wire cutting machine. Mercury had a surface tension of 485 dynes/cm, a contact angle of 130°, and an applied pressure range of 0.1 to 60,000 psia.

#### 2.3.7. Fibre Dispersion

The fluorescence technique-based method [40] was used to evaluate the dispersion coefficients of PVA fibres. Cross-sectional samples with a dimension of 70.7 mm × 70.7 mm were cut from the mortar specimens. Images of the samples’ fluorescence were captured using a CCD camera. In order to count the PVA fibres, these images were divided into unit sections via image analysis tools. The PVA fibres’ dispersion coefficient was calculated based on the count per unit area.

#### 2.3.8. Microstructure

To analyse the geopolymer matrix’s microstructure, scanning electron microscopy (SEM) was carried out. Samples were cut from the mortar specimens using a diamond wire-cutting machine. To avoid an excessive charge reflection, they were gold-coated.

## 3. Results and Discussion

### 3.1. Flowability

**Figure 5:**
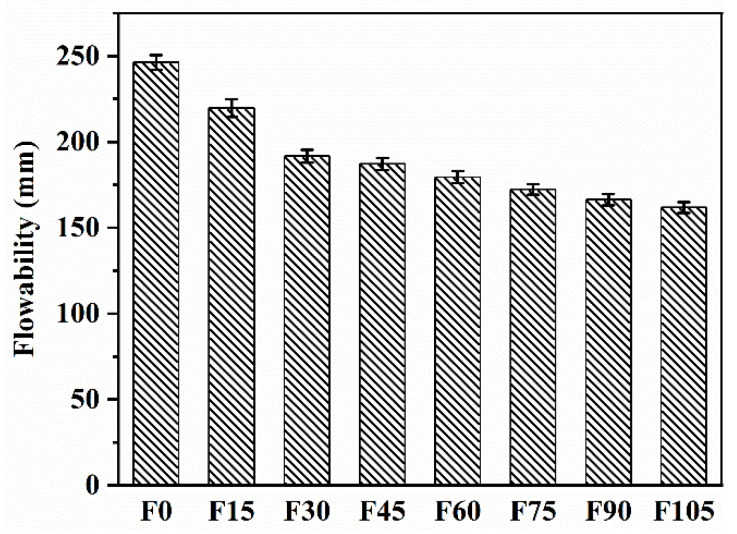
Flowability of F0–F105.

Figure 5 shows the flowability of F0–F105. The flowability of F15–F105 decreased by 10.78%, 22.17%, 23.98%, 27.11%, 30.01%, and 34.28%, respectively, compared with that of F0. The flowability decreased as the volume fractions of PVA fibres increased. The phenomenon has also been reported for cement mortar containing 100% river sand [41]. Several factors were blamed for the decreased flowability. The PVA fibres made it difficult for the fresh geopolymer paste to move. The difficulty was made worse by the hydrophilicity and high aspect ratio of PVA fibres as well as the high viscosity of the geopolymer paste. The values of flowability of F15–F105 (161.85–219.72 mm) nevertheless meet the standards for construction even if the flowability was decreased by the addition of PVA fibres.

### 3.2. Compressive Strength

**Figure 6:**
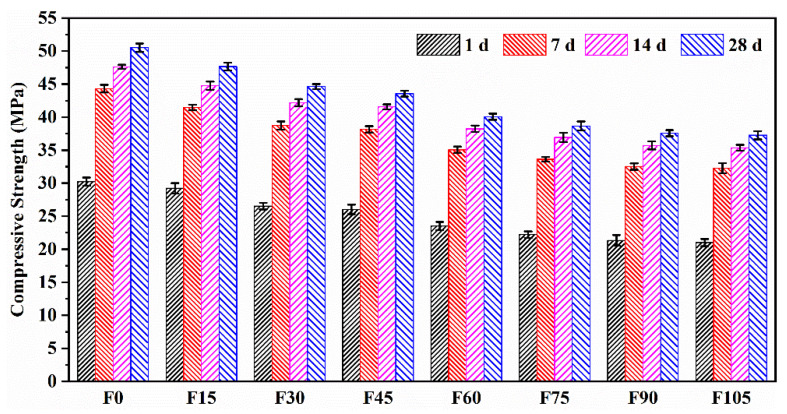
Compressive strengths of F0–F105.

Figure 6 shows the compressive strengths of F0–F105. Their 1 and 7 d compressive strengths were 56.39–61.36% and 86.54–87.72% of the 28 d one, respectively, suggesting that the geopolymer mortar can achieve a high early compressive strength without high-temperature curing (60 °C). The high early compressive strength is an advantage of the geopolymer mortar over conventional cement mortar. It is worth noting that a sufficiently high early strength is desirable during engineering construction because of decreased construction periods.

The 28 d compressive strengths of F15–F105 reduced by 7.50%, 13.36%, 15.44%, 22.21%, 24.91%, 27.03%, and 27.65%, respectively, compared with that of F0. This indicates that the compressive strength was decreased by the addition of PVA fibres, which was attributed to the increased porosity. Similarly, Hossain et al. and Mohammed et al. stated that the incorporation PVA fibres caused a decrease in the compressive strengths of concrete [42] and engineered cementitious composites [43] owing to the increased porosity. Furthermore, the reductions in compressive strengths of F15–F45 were lower than those of F60–F105. This trend can be explained by the dispersion of PVA fibres. The dispersion coefficients of F15, F30, and F45 remained high, whereas those of F60–F105 exhibited an obvious reduction. The poorer dispersion of PVA fibres of F60–F105 caused their higher reductions in the compressive strength. In addition, the incorporation of PVA fibres significantly reduced the flowability, which adversely affected the compactness of the geopolymer mortar and contributed to the loss of compressive strength. A similar explanation for the declined compressive strength of cement mortar containing polypropylene fibres was given by Puertas [44].

Although the compressive strength was decreased by the addition of PVA fibres, the values of F0–F105 (37.26–50.50 MPa) are still high enough for their use as binders in the production of normal- and high-strength concrete. The slight reductions in compressive strengths of F15, F30, and F45 showed that the incorporation of 0.15–0.45 vol.% of PVA fibres had little impact on the geopolymer mortar’s compressive strength.

### 3.3. Flexural Strength

**Figure 7:**
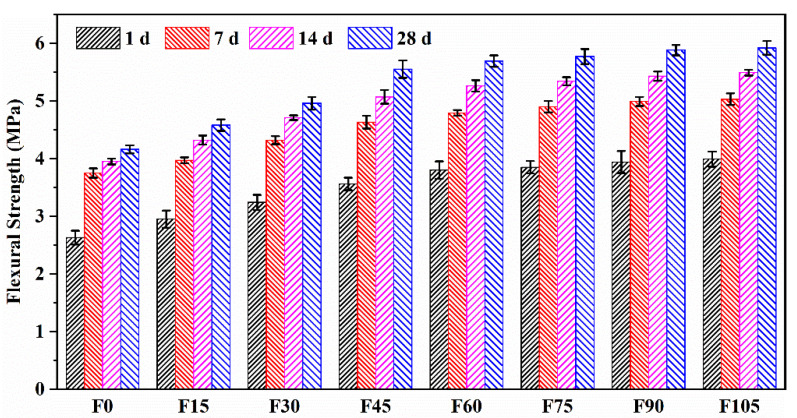
Flexural strengths of F0–F105.

Figure 7 shows the flexural strengths of F0–F105. Their 1 and 7 d flexural strengths were 63.22–67.40% and 83.42–90.14% that of the 28-d one, respectively, suggesting that the geopolymer mortar can attain a high level of early flexural strength. This trend agreed with that of compressive strength, but it was opposite to the PVA fibre content.

The increased PVA fibre content resulted in an increase in the flexural strength. The 28 d flexural strengths of F15–F105 increased by 10.10%, 19.23%, 33.41%, 36.78%, 38.70%, 41.35%, and 42.31%, respectively, compared with that of F0. This suggests that the addition of PVA fibres improved the geopolymer mortar’s flexural strength, which benefitted from the bridging effect of PVA fibres. Similarly, Xu et al. [24] reported the improved toughness for PVA fibre-reinforced FA-based geopolymer composites. In addition, the improvement was significant when PVA fibre content exceeded 4.5 vol%. Although F60–F105 exhibited a small improvement in the flexural strength relative to F45, the ECO_2_e and EE of F60–F105 were higher than those of F45 owing to the increased content of PVA fibres. In this regard, the incorporation of 4.5 vol% of PVA fibres achieved an optimal balance between improvement in flexural strength and eco-efficiency.

### 3.4. Drying Shrinkage

**Figure 8:**
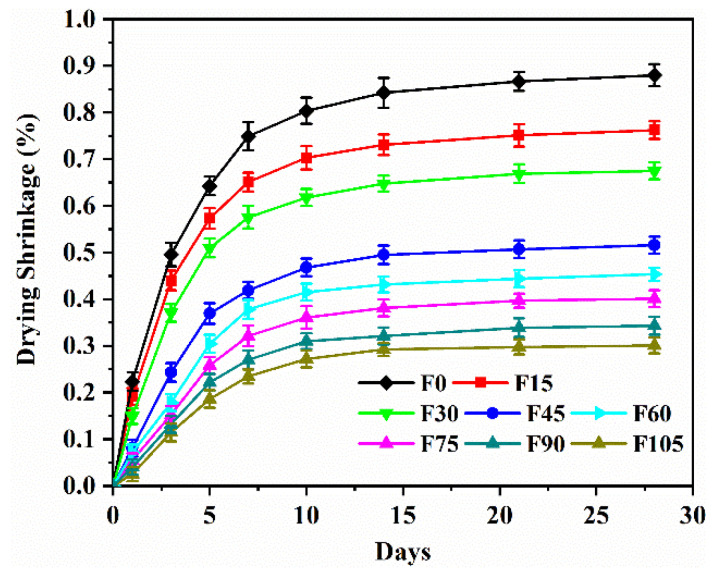
Drying shrinkage of F0–F105.

Figure 8 shows the drying shrinkage of F0–F105. The drying shrinkage increased with the curing ages, and it increased significantly before 7 d but relatively slowly between 7 and 28 d. It is worth noting that the precursors included GBFS and FA. Because GBFS had higher reactivity than FA, GBFS was activated by alkalis at an early age to produce the majority of the reaction products. The reaction products showed a self-desiccation, and the amount of capillary free water decreased, which caused the loss of internal moisture and resulted in the rapid drying shrinkage before 7 d [45,46]. Furthermore, FA had a dilution effect on GBFS, and the rate of alkaline activation decreased, which explains why the increase in drying shrinkage was lower between 7 and 28 days [47,48]. Deng et al. [33] reported similar results for FA and GBFS-blended geopolymer mortar containing 100% river sand.

F0 exhibited a significantly high drying shrinkage, and its drying shrinkage at 28 d was 0.88%. The 28 d drying shrinkage of cement mortar containing 20% WOS aggregates was about 0.048–0.061% [1], whereas that of the geopolymer mortar was 0.88%. The geopolymer mortar in this study exhibited a much higher drying shrinkage than the cement mortar. Three factors were blamed for the increased drying shrinkage of the geopolymer mortar. First, it is well known that the drying shrinkage of geopolymer is greater than that of hydrated cement. This accounts for the geopolymer mortar’s greater drying shrinkage than cement mortar. Second, WOS aggregates have a higher water absorption than river sand [1], which reduces the amount of capillary free water retained in matrix. This causes a higher loss of capillary free water and results in a higher drying shrinkage of the mortar containing WOS aggregates than that containing 100% river sand. Kuo et al. [49] also reported that a higher percentage of river sand replacement with WOS aggregates led to higher drying shrinkage. Third, WOS aggregates have a lower Young’s modulus than natural fine aggregates such as river sand [50]. Specimens containing the high-Young’s modulus aggregates show a higher drying shrinkage than the low-Young’s modulus ones [49].

The 28 d drying shrinkage of F15–F105 declined by 13.37%, 23.31%, 41.36%, 48.45%, 54.41%, 60.97%, and 65.79%, respectively, indicating that the inclusion of PVA fibres significantly controlled the geopolymer mortar’s drying shrinkage. The inhibitory effect on the drying shrinkage was credited to PVA fibres’ bridging effect. The drying shrinkage-induced microcracks were bridged by PVA fibres, which also controlled the spread of the cracks. Moreover, the PVA fibre–geopolymer matrix interface has a good bonding owing to their strong chemical bonding, which maximises the bridging effect [51]. The decrease in drying shrinkage that benefitted from the inclusion of PVA fibres was also found in geopolymer composites [29,30,31], cementitious composites [43], and concrete [52].

### 3.5. Sorptivity

**Figure 9:**
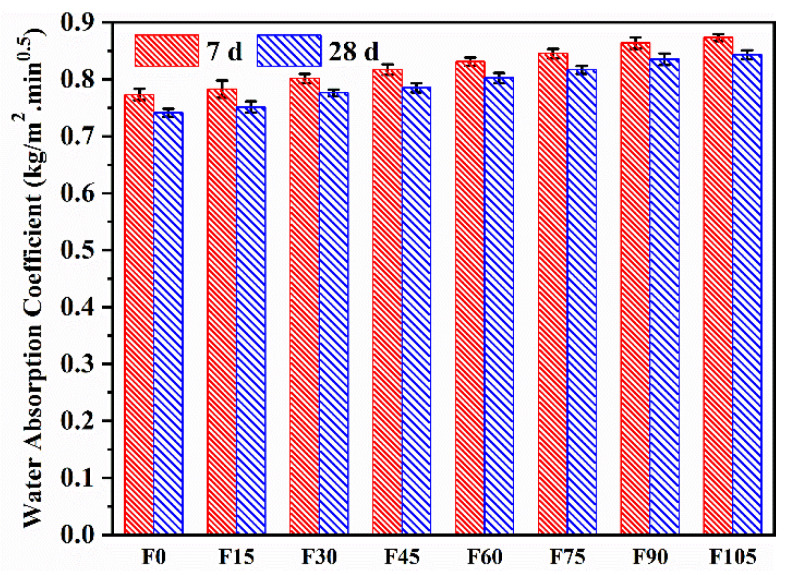
Water absorption coefficients of F0–F105.

The water absorption coefficients of F0–F105 are shown in Figure 9. The water absorption coefficients of F15–F105 were higher than that of F0, indicating that the incorporation of PVA fibres led to an increase in the sorptivity of the geopolymer mortar, and the sorptivity increased with the increasing content of PVA fibres. The higher porosity was blamed for the increase in sorptivity. Similarly, Shah et al. [53] and Abdollahnejad et al. [54] stated that the incorporation of PVA fibres introduced pores and cavities into the geopolymer mortar containing 100% river sand. Moreover, PVA fibres are hydrophilic (Table 2) and readily absorb water, which caused an increase in sorptivity. On the other hand, the 28 d water absorption coefficients were lower than the 7 d ones owing to the densification of the microstructure as the AAR proceeded [33]. The 28 d water absorption coefficients of F15–F105 increased by 1.32%, 4.68%, 5.91%, 8.21%, 10.17%, 12.65%, and 13.73%, respectively, compared with that of F0. Although the water absorption coefficients increased with increasing PVA fibre content, the increase amplitude was slight when its content was lower than 4.5 vol%. The slight increase in sorptivity is still acceptable.

### 3.6. RCP

**Figure 10:**
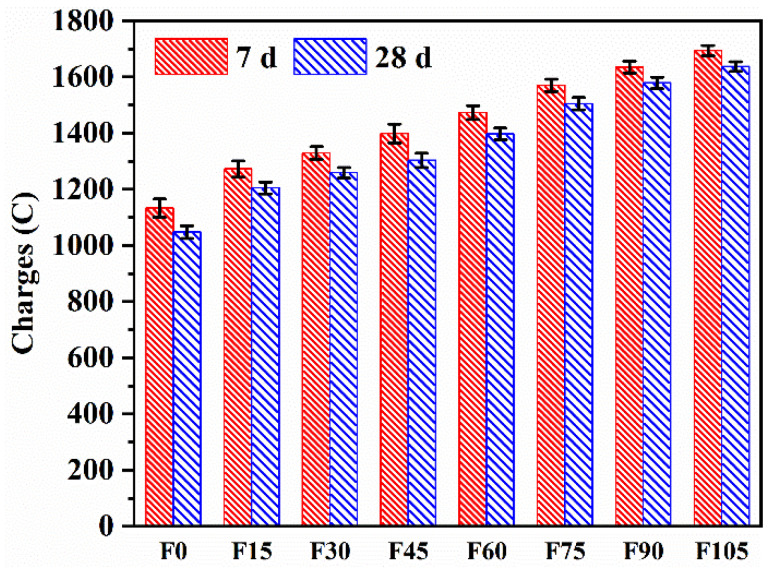
Total charges passed of F0–F105.

The total charges passed of F0–F105 are shown in Figure 10. The RCP change trend was similar to that of sorptivity. The 28 d charges were lower than the 7 d ones owing to the denser microstructure as the AAR proceeded [33]. The 28 d total charges passed of F15–F105 increased by 15.09%, 20.25%, 24.45%, 33.43%, 43.74%, 50.81%, and 56.35%, respectively, compared to those of F0, demonstrating that the chloride resistance was dramatically reduced by the addition of PVA fibres, which was attributed to the increased porosity. The decreased chloride resistance was also found in the geopolymer mortar containing 100% river sand, with the same cause [33]. However, the total charges passed of F0–F105 were between 1000 and 2000 C, indicating that they are categorised as low chloride penetrability based on ASTM C1202-19 [55]. The densified microstructure enhanced by C-A-S-H [56] and the increased chloride binding enhanced by N-A-S-H [57] resulted in the geopolymer mortar’s high chloride resistance.

### 3.7. Porosity

**Figure 11:**
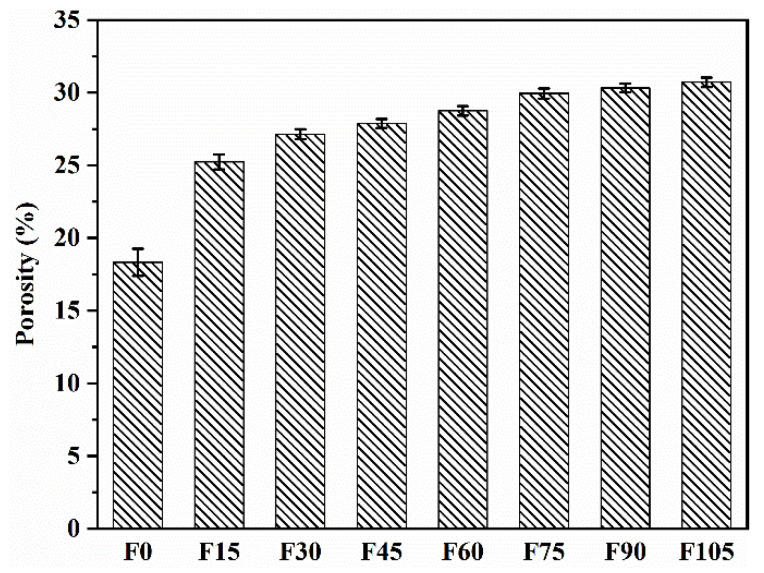
Porosities of F0–F105.

The porosity values of F0–F105 are shown in Figure 11. Their values are 18.32%, 25.24%, 27.14%, 27.88%, 28.75%, 29.94%, 30.33%, and 30.71%, respectively, suggesting that the porosity was increased by the addition of PVA fibres. The geopolymer mortar containing 100% river sand was likewise found to have an increase in porosity brought on by the addition of PVA fibres [33]. Although the porosity increased as the PVA fibre content increased, the porosities of F30–F105 were 1.9%, 2.64%, 3.51%, 4.70%, 5.09%, and 5.54% higher than that of F15, respectively. These values showed that the increase in porosity was slight, especially for the PVA fibre content of less than 4.5 vol%.

### 3.8. Fibre Dispersion

**Figure 12:**
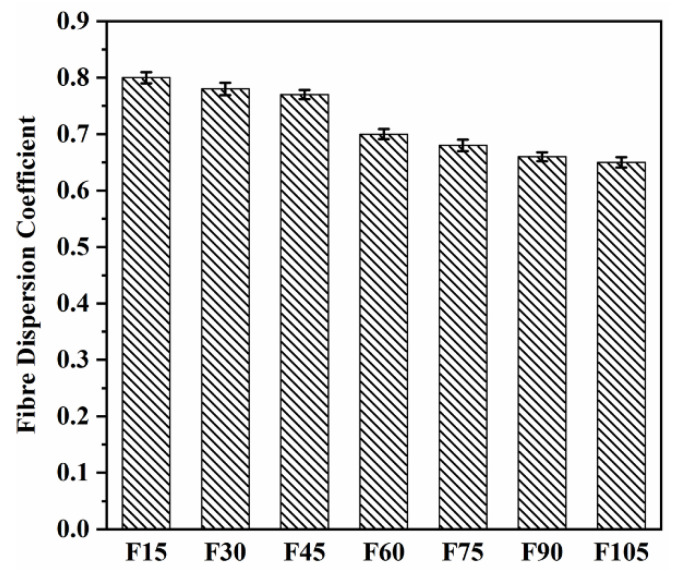
Fibre coefficients of F15–F105.

The dispersion coefficients of PVA fibres of F15–F105 are shown in Figure 12. A good (uniform) fibre dispersion is indicated by a dispersion coefficient value that is close to 1.0, whereas a poor fibre dispersion is indicated by a value that is close to 0. As the PVA fibre content increased, the dispersion coefficients decreased. PVA fibres have a large surface area as well as strong hydrogen bonds on their surfaces, which were responsible for the decreased dispersion coefficients [58]. In addition, it can be observed that the dispersion coefficients of F15–F45 were in a small range (0.77–0.80), and those of F30 and F45 slightly declined by 2.50% and 3.75%, respectively, compared with that of F15, indicating that F15–F45 did not exhibit a significant decrease in the dispersion coefficients. However, the dispersion coefficients of F6–F105 decreased by 12.5%, 15%, 17.5%, and 18.75%, respectively, compared with that of F15. F6–F105 showed a relatively larger reduction in the dispersion coefficients than F30 and F45, which was due to the larger number of PVA fibres for F6–F105. The compressive strength had the same trend.

### 3.9. SEM

**Figure 13:**
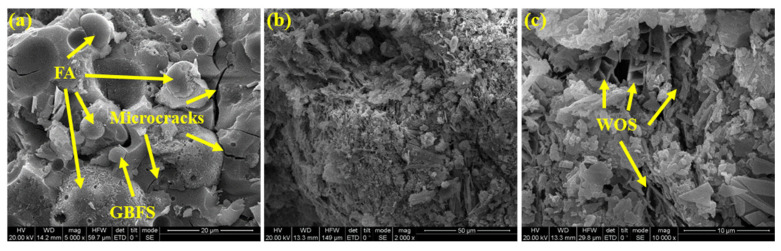
SEM micrographs of geopolymer matrix (**a**), amorphous morphology (**b**), and WOS (**c**).

The micrographs of the geopolymer matrix are shown in Figure 13a. The drying shrinkage led to the microcracks that were seen in the geopolymer matrix. Moreover, spherical and polygonal particles were observed. The spherical ones were raw FA particles, whereas the polygonal ones were raw GBFS particles. The surface of the spherical particles showed a rough texture, indicating that the FA particles partially reacted in the AAR. Spherical trajectories were also observed because the particles separated from the geopolymer matrix. Steveson and Sagoe-Crentsil [59] stated similar observations and explained that due to the weak interfacial interaction, FA particles were vulnerable to separating from the polymerised aluminosilicate gels. In addition, Figure 13b shows an amorphous morphology. Figure 13c shows WOS aggregates, and they have a layered microstructure.

### 3.10. Eco-Efficiency

**Figure 14:**
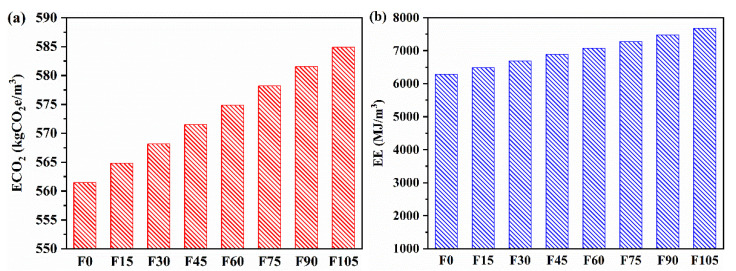
ECO_2_e (**a**) and EE (**b**) of F15–F105.

The embodied CO_2_ emissions (ECO_2_e) and embodied energy (EE) of raw materials are shown in Table 4. Based on these values and the mix proportions (Table 3), the ECO_2_e and EE of F0–F105 were calculated, and Figure 14 shows the results. The ECO_2_e of F15–F105 increased by 0.60%, 1.19%, 1.79%, 2.38%, 3.00%, 3.57%, and 4.17%, respectively, compared with that of F0. Although the addition of PVA fibres caused an increase in ECO_2_e, the amplitudes of the increases were minor. The EE of F15–F105 increased by 3.13%, 6.27%, 9.40%, 12.54%, 15.67%, 18.81%, and 21.94%, respectively, compared with that of F0. The increased amplitudes of EE were significantly higher than those of ECO_2_e, which were attributed to the high EE of PVA fibres relative to other raw materials. However, the increase in EE was still limited when less than 4.5 vol% of PVA fibres were adopted.

Furthermore, the ECO_2_e of conventional geopolymer mortar containing 100% river sand was 634.16 kgCO_2_e/m^3^ [60], and that of F0–F105 was 561.48–584.88 kgCO_2_e/m^3^. These values indicate that the geopolymer mortar containing WOS aggregates and 0–1.05 vol% of PVA fibres had a lower CO_2_ emission than the conventional one containing 100% river sand. The EE of the conventional geopolymer mortar was 6957.44 MJ/m^3^ [60], whereas that of F0–F45 was 6293.08–6884.81 MJ/m^3^, and that of F60–F105 was 7082.05–7673.78 MJ/m^3^. These values indicate that the geopolymer mortar with a PVA fibre content higher than 4.5 vol% caused a higher energy consumption than the conventional one, but the PVA fibre content lower than 4.5 vol% still resulted in lower energy consumption than the conventional geopolymer mortar. Therefore, the PVA fibre content should not be higher than 4.5 vol% in the production of the geopolymer mortar containing WOS aggregates to achieve a high eco-efficiency.

**Table 4:** ECO_2_e and EE of Raw Materials (per kg).

Raw Materials		ECO_2_e (kgCO_2_e)	EE (MJ)	References
Activators	SS	1.237	10.20	[61]
	SH	0.7458	20.50	[61]
Precursors	GBFS	0.5112	1.60	[61]
	FA	0.008	0.10	[62]
Fine aggregates	Sand	0.002796	0.081	[61]
	WOS	0	0	[3]
Fibres	PVA	1.71	101.00	[63]
Processing		0.0038	0.15	[61]

## 4. Conclusions

Recycling WOS aggregates in geopolymer mortar has not been reported. The influence of PVA fibres on the engineering properties of the new geopolymer mortar is still unclear. To bridge the gap, this study investigated the influence of various PVA fibre contents (0–1.05 vol%) on its flowability, compressive, flexural strength, drying shrinkage, sorptivity, chloride resistance, porosity, fibre dispersion, ECO_2_e, and EE. The following conclusions can be drawn.

(1) Due to the restriction of the fresh geopolymer paste’s free flow, the flowability was decreased by the addition of 0.15–1.05 vol% of PVA fibres, but the flowability (161.85–219.72 mm) still complies with construction standards. The inclusion of PVA fibres also caused a reduction in compressive strength because of the increased porosity, decreased fibre dispersion, and decreased compactness. However, the compressive strengths at 28 d for 0.15–0.45 vol% of PVA fibres were 47.64–43.55 MPa, which were sufficient enough for construction. As a result of PVA fibres’ bridging effect, the flexural strengths for 0.15–1.05 vol% of PVA fibres rose by 10.10–42.31%.

(2) The geopolymer mortar had good durability. The drying shrinkage at 28 d for 0.15–1.05 vol% of PVA fibres declined by 13.37–65.79% because of PVA fibres’ bridging effect. The chloride resistance for 0.15–1.05 vol% of PVA fibres was classified as the low chloride penetrability, owing to the good chloride binding of AAR products. Moreover, when the dosage was less than 4.5 vol%, the sorptivity increased just slightly.

(3) The geopolymer mortar had good eco-efficiency. The ECO_2_e and EE for the PVA fibre content less than 0.45 vol% were lower than those of conventional geopolymer mortar containing 100% river sand. The optimum content of PVA fibre is suggested to be 0.45 vol% because it concurrently maximises the engineering properties and eco-efficiency. The geopolymer mortar is recommended for subtropical monsoon climates.

(4) The cost analysis and high-temperature resistance of the geopolymer mortar are neglected in this study, and they should be evaluated in future work. This study can be expanded using machine learning techniques based on the references [64,65].

## Figures and Tables

**Table 1:** Chemical Constituents of FA and GGBFS (wt%).

Constituents	FA	GBFS
Fe_2_O_3_	0.42	6.49
CaO	5.2	38.23
MgO	2.35	5.66
Al_2_O_3_	26.98	14.01
SiO_2_	61.54	35.1
Na_2_O	0.2	0.1
K_2_O	0.15	0.07
TiO_2_	0.05	-
MnO	-	0.17
LOI	2.88	0.49

**Table 2:** Characteristics of PVA Fibres.

Characteristics	Values
Tensile strength (MPa)	1466.55
Modulus of elasticity (GPa)	36.98
Elongation at dry breaking (%)	7
Length (mm)	12 ± 0.5
Diameter (μm)	200
Resistance to hot water (°C)	≥104
Specific weight	1.3
Water contact angle (°)	81.5

**Table 3:** Mix Proportions of F15–F105.

Mix ID	FA (kg/m^3^)	GBFS (kg/m^3^)	SH (kg/m^3^)	SS (kg/m^3^)	Sand (kg/m^3^)	WOS (kg/m^3^)	PVA Fibre (vol%)
F0	481	207	138	276	880	220	0
F15	481	207	138	276	880	220	0.15
F30	481	207	138	276	880	220	0.30
F45	481	207	138	276	880	220	0.45
F60	481	207	138	276	880	220	0.60
F75	481	207	138	276	880	220	0.75
F90	481	207	138	276	880	220	0.90
F105	481	207	138	276	880	220	1.05

## Data Availability

The data presented in this study are available on request from the corresponding author.
